# Developing In Vitro–In Vivo Correlation for Bicalutamide Immediate-Release Dosage Forms with the Biphasic In Vitro Dissolution Test

**DOI:** 10.3390/pharmaceutics17091126

**Published:** 2025-08-28

**Authors:** Nihal Tugce Ozaksun, Tuba Incecayir

**Affiliations:** Department of Pharmaceutical Technology, Faculty of Pharmacy, Gazi University, Etiler, 06330 Ankara, Turkey; tugceozaksun@gazi.edu.tr

**Keywords:** biphasic in vitro dissolution, in vitro–in vivo correlation (IVIVC), bicalutamide

## Abstract

**Background/Objectives**: Reflecting the interaction between dissolution and absorption, the biphasic dissolution system is an appealing approach for estimating the intestinal absorption of drugs in humans. The study aims to characterize the suitability of the biphasic in vitro dissolution testing to set up an in vitro–in vivo correlation (IVIVC) for the original and generic immediate-release (IR) tablets of a Biopharmaceutics Classification System (BCS) Class II drug, bicalutamide (BIC). **Methods**: USP apparatus II paddle was used to conduct dissolution testing. A level A IVIVC was obtained between in vitro partitioning and in vivo absorption data of the original drug. The single-compartmental modeling was used for pharmacokinetic (PK) analysis. The generic product’s plasma concentrations were estimated. **Results**: There was a good correlation between in vitro and in vivo data (*r*^2^ = 0.98). The area under the concentration–time curve (*AUC*) and maximum plasma concentration (*C_max_*) ratios for generic/original were 1.04 ± 0.01 and 0.951 ± 0.026 (mean ± SD), respectively. **Conclusions**: The biphasic dissolution testing may present an in vivo predictive tool for developing generic products of poorly soluble and highly permeable drugs such as BIC, which are characterized by pH-independent poor solubility.

## 1. Introduction

In vitro dissolution studies are vital for drug formulation development and the quality control of drug products. In addition, they are significant for establishing in vitro–in vivo correlations (IVIVC) to anticipate in vivo performance of the products [1,2]. Dissolution tests are frequently performed using the compendial dissolution equipment and methods. However, the compendial equipment and methods have low discriminatory power and use conditions that limit their ability to mimic specific features of in vivo solubility and dissolution of drugs [3]. The in vivo dissolution and absorption of oral drugs are significantly influenced by the human gastrointestinal (GI) tract’s physiological parameters, such as pH, volume of fluid, bile salt, and GI transit time [4]. Thus, it is essential to establish biorelevant dissolution methods to identify the in vivo solubility–absorption interplay and forecast the drug products’ in vivo performance in humans [5].

Reflecting the interaction between dissolution and absorption, the use of biphasic dissolution systems is an appealing approach for precisely estimating the in vivo behavior of drugs in humans. The system contains the aqueous (buffer) and organic phases (octanol) representing the drug’s dissolution and absorption processes, respectively [6]. Following the dissolution in the buffer, the drug partitions into the organic solvent medium based on its lipophilicity, yielding a more realistic drug supersaturation in the buffer medium and maintaining sink conditions in the organic solvent. Therefore, it enables the combined evaluation of drug dissolution and partition kinetics [7]. A wide range of non-polar solvents has been used in the biphasic dissolution studies [8,9,10,11]. Among these solvents, octanol is considered to be the best [12], since it is poorly soluble in water (0.5 g/L), has a low density (0.83 g/cm^3^ at 20 °C), enables easier sampling, and does not evaporate at 37 °C, keeping the upper phase volume fixed [13].

Pioneering studies on biphasic dissolution started in 1967 and were conducted to study the partitioning of benzoic acid and salicylic acid tablets [8,14]. Stead et al. were the first to investigate the correlation between the biphasic in vitro and in vivo outcomes of ibuprofen formulations, demonstrating a promising IVIVC [15]. In the past five decades, various biphasic dissolution systems such as a modified rotating basket, a basket–paddle hybrid system, a flow-through cell coupled with a basket, and a miniaturized system have been developed to study the dissolution and absorption kinetics of model drugs which have poor water solubilities [11,13,15,16,17]. In the meantime, studies have demonstrated the promising potential of biphasic dissolution tests in discriminating various formulations and conducting IVIVCs [16,17,18].

The present study aims to develop an IVIVC for bicalutamide (BIC) immediate-release (IR) dosage forms with the biphasic in vitro dissolution test. BIC, a non-steroidal antiandrogen, is used to treat prostate cancer. It prevents the stimulatory effects of androgens on prostate cancer cells [19]. The R-enantiomer of BIC is primarily responsible for its pharmacological activity and is predominant in human plasma. The R-enantiomer reaches the maximum plasma concentration (*C_max_*) of 559–970 ng/mL within 15 to 48 h after administration of a single dose of BIC (50 mg) in healthy males. The mean elimination half-life (*t*_1/2_) is 4.2 days. On the contrary, the S-enantiomer reaches *C_max_* of 32–66 ng/mL within 2 to 5 h post administration and exhibits a mean *t*_1/2_ of 19 h [20,21]. BIC’s oral absorption is slow due to the enterohepatic circulation. However, it is extensively absorbed after oral administration based on the total radioactivity excreted in urine (36%) and feces (43%) in humans receiving a single oral dose (50 mg), as extensive metabolism necessitates extensive absorption. Additionally, the absolute bioavailability (BA) data are absent in humans since the intravenous formulation is not available [20,21]. Oral absorption was also found to be about 80% in rat, mouse, rabbit, and dog [22]. Glucuronidation and hydroxylation followed by glucuronidation are the primary metabolic pathways for the S- and R- enantiomers, respectively. Fecal excretion points out the secretion of glucuronide conjugates in bile and subsequent hydrolysis in the GI tract [20,21,23]. Thus, BIC can be classified as a Class II drug (low solubility/extensive metabolism) according to the Biopharmaceutics Drug Disposition Classification System (BDDCS) that categorizes drugs based on their solubility and overall extent of metabolism in humans [24,25].

This study characterizes the suitability of the biphasic in vitro dissolution testing to set up an IVIVC for the original and generic IR tablets of BIC (CAS 90357-06-5). It assesses the significance in forecasting the drug’s BA in humans. BIC is a low solubility/high permeability drug (Biopharmaceutics Classification System (BCS) Class II) [26]. Displaying dissolution rate limited absorption, BCS Class II drugs are good candidates for developing IVIVCs. The plasma drug concentration profile following the oral administration of the generic formulation in healthy male volunteers was digitized from a previously published paper [21]. An excellent relation was established between in vitro biphasic dissolution and in vivo absorption data. A predictive model-dependent strategy based on this correlation was identified to forecast in vivo human results of BIC’s generic formulation.

## 2. Materials and Methods

### 2.1. Materials

BIC was kindly given by Onko & Koçsel Pharmaceuticals (Istanbul, Turkiye). Sodium chloride, sodium hydroxide, hydrochloric acid, 1-octanol, monopotassium phosphate, and sodium lauryl sulfate (SLS) were purchased from Sigma-Aldrich (Steinheim, Germany). The original and the generic 50 mg BIC IR tablets were bought from a local drug store. The commercial original (reference) (Casodex^®^, license holder: AstraZeneca, manufacturer: CordenPharma, Plankstadt, Germany, batch number: D21254A, serial number: 60000019352101) and the generic (test) (batch number: 30739410, serial number: BDDW177E) tablets were utilized. All the chemicals used were of analytical grade.

### 2.2. Single-Phase Dissolution Test

The compendial test was performed using USP apparatus II (Agilent Technologies 708-DS, Petaling Jaya, Malaysia) for the original and generic products at 37 °C. The dissolution media were 1000 mL water containing 1% SLS and phosphate buffer (50 mM, pH 6.8) for the sink and non-sink conditions, respectively. The paddle’s rotation speed was 50 rpm.

The samples were withdrawn at certain times and filtered by a Chromafil^®^ syringe filter (CA45/25, 0.45 µm, Eschau, Germany), and followed by the addition of an equal volume of freshly prepared medium. The spectrophotometric method was used to determine the concentrations of BIC. The experiments were conducted in a total of six repeats. The total percentage (mean ± standard deviation (SD)) was calculated and represented graphically against time.

### 2.3. Biphasic Dissolution Test

The biphasic testing was performed as previously detailed [27]. Briefly, the aqueous (pH 6.8 phosphate buffer, 300 mL) and organic phases (octanol, 200 mL) were saturated through stirring (50 rpm) for 45 min at 37 °C. A tube extended into the octanol was employed to discharge the tablets into the aqueous phase. Thus, the tablets were avoided from being in contact with octanol. The amount of octanol (200 mL) provided the sink condition based on the saturation solubility of BIC in octanol (2.13 × 10^−3^ mol/L at 35 °C) [28].

**Figure 1:**
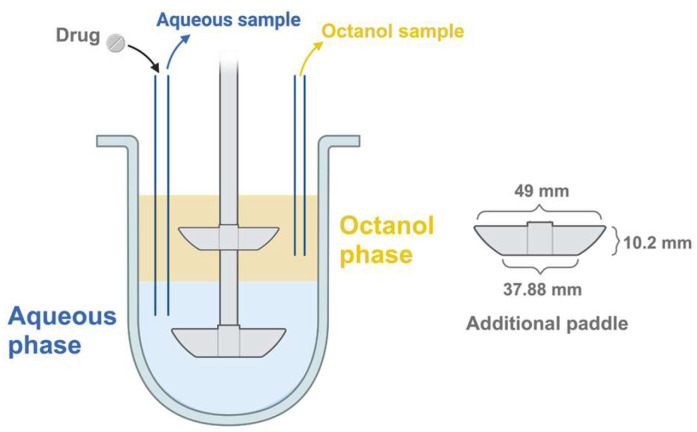
The vessel of the USP apparatus II with the additional paddle.

The second paddle was placed in the middle of the octanol phase to ensure enough mixing. The primary paddle was fully immersed in the aqueous medium (300 mL). The samples (5 mL) were simultaneously collected from the phosphate buffer and octanol phases at 15, 30, 45, 60, 90, 120, 180, and 240 min. These samples were subsequently filtered using a Chromafil^®^ syringe filter (CA45/25, 0.45 µm). Each sampling was followed by the addition of an equal volume of fresh medium to maintain sink conditions. The spectrophotometric method was employed to analyze BIC concentration in the samples. The experiments were conducted in triplicate. The cumulative percentage of drug (mean ± SD) in both phases was plotted against time. The system configuration is displayed in Figure 1.

### 2.4. Analytical Method

Analysis was carried out spectrophotometrically with a Cary 60 UV-Vis spectrophotometer (Agilent Technologies, Santa Clara, CA, USA). Absorbance for octanol, pH 6.8 phosphate buffer, and water containing 1% SLS was measured at 272, 273, and 272 nm, respectively. The calibration curves for the corresponding medium were used to determine the concentration of BIC in the samples. High linearity (*r*^2^ = 0.999) was achieved within the 3–18 μg/mL calibration range. The range of accuracy was between 98.9% and 102%. The precision was less than 1% (SD). The quantification limit (QL) vs. detection limit (DL) were 2.1 vs. 0.7 µg/mL, 0.08 vs. 0.03 µg/mL, and 0.11 vs. 0.04 µg/mL in octanol, pH 6.8 phosphate buffer, and water containing 1% SLS, respectively.

### 2.5. Prediction of Human Plasma Profiles with the Biphasic Dissolution Data

The fraction of drug dose absorbed (*F_abs_*) values were calculated using the published single-dose pharmacokinetics (PK) data of the original drug in healthy males [21]. *F_abs_* values were calculated using the Wagner-Nelson method (Equation (1)) [29].

(1) $$F_{abs}=\frac{k_{d}\int_{0}^{t}C\left(t\right)dt+C\left(t\right)}{k_{d}\int_{0}^{\infty }C\left(t\right)dt}\;\;\;\;\;\;\;\;\;\;\;\;\;\;\;\;\;\;\;\;\;\;\;\;\;$$

where *C(t)* is the plasma concentration of the drug (ng/mL), and *k_d_* is the elimination rate constant (h^−1^).

The published plasma profile of the original drug was digitized using the Automeris.io V5 software to determine the in vivo time points (0–672 h) for the plasma profiles. For each healthy male volunteer, the plasma profiles of the original drug were obtained using Equation (2) with the PK parameters derived from the literature data (Table 1) [21].

A Level A IVIVC was established between in vitro dissolution (fraction of BIC partitioned into octanol at 15, 30, 45, 60, 90, 120, 180, and 240 min) and in vivo absorption data (*F_abs_* at 1, 2, 3, 5, 7, 9, 12, and 15 h) for the reference. Levy’s plot was drawn to assess the time scaling. Thus the correlation between in vitro (dissolution) and in vivo (absorption) time points was assessed. Linear regression was used to investigate the relationship between variables in Microsoft 365 Excel. The Level A IVIVC was used to estimate the *F_abs_* values of healthy male volunteers for the generic formulation. Subsequently, the absorption rate constant (*k_a_*) of each volunteer was determined by the Wagner-Nelson method for the generic [30]. The single-compartmental modeling was used for PK analysis. The determination coefficient (*r*^2^) of the one compartment model was 0.990 for the observed mean profile of the original drug (reference). The generic product’s plasma concentrations (*C_p_*) were estimated using Equation (2), with the *k_a_*, elimination rate constant (*k_d_*), and volume of distribution (*V_d_*) PK parameters specific to each subject. The area under the plasma concentration–time curve from 0 to infinity (*AUC*_0→∞_*)* was determined using the trapezoidal rule method.

(2) $$C_{p}=\frac{FF^{*}D}{V_{d}}\;\frac{k_{a}}{\left(k_{a}-k_{d}\right)}\;\left(e^{{-k}_{d}t}-e^{{-k}_{a}t}\right)$$

where FF* is bioavailability constant, *D* is dose (ng), *V_d_* is volume of distribution (mL), *k_a_* is absorption rate constant (h^−1^), *k_d_* is elimination rate constant (h^−1^), and *t* is time (h). 

### 2.6. Data Analysis

All data were expressed as mean ± SD. The similarity of dissolution profiles was assessed by the *f*_2_ test [31]. The *f*_2_ value was determined using Equation (3):

(3) $$f_{2}=50\log\frac{100}{\sqrt{1+\frac{1}{n}\sum_{t=1}^{n}{\left(R_{t}-T_{t}\right)}^{2}}}$$

where *n* is number of samples, *R_t_* and *T_t_* are cumulative dissolution percentages of the reference and test products at the specific time point, respectively. The calc *f*_2_ > 50 points outs the similarity of the reference and test profiles.

## 3. Results

### 3.1. Single-Phase Dissolution Test

**Figure 2:**
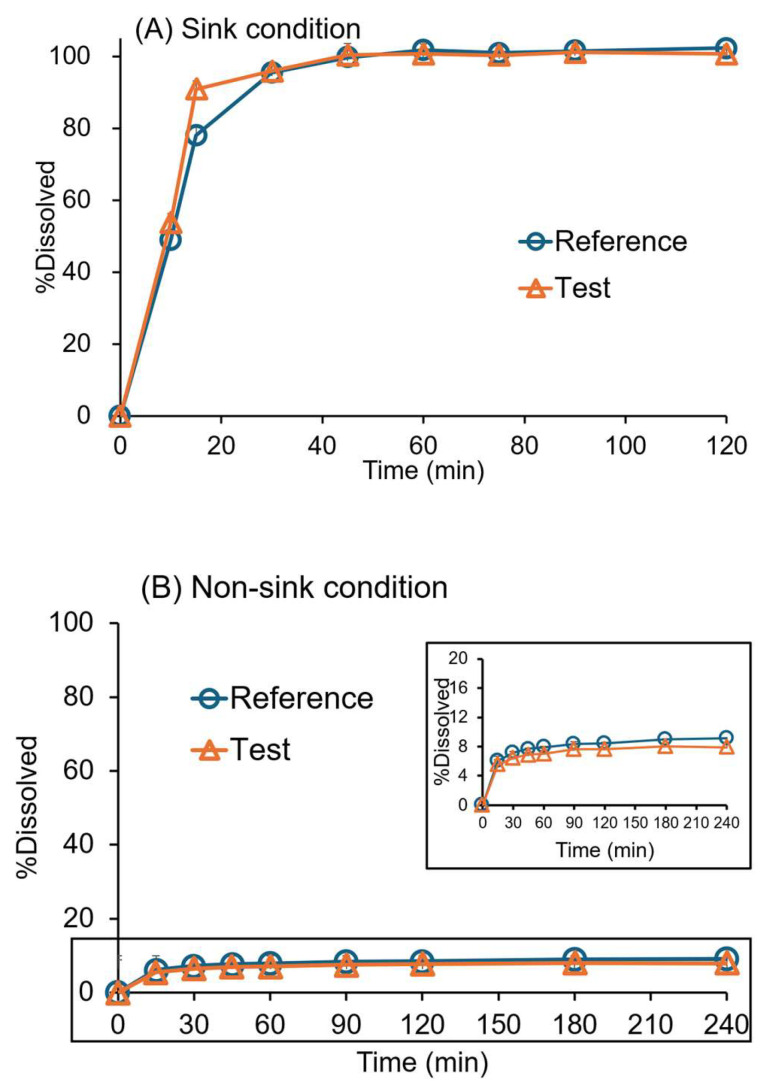
Dissolution profiles of 50 mg bicalutamide (BIC) original (reference) and generic (test) tablets in a single-phase dissolution test using the USP paddle (rotation speed = 50 rpm; 37 ± 0.5 °C; mean ± SD; *n* = 6). (**A**) Sink condition (water with 1% SLS, 1000 mL) and (**B**) non-sink condition (pH 6.8 phosphate buffer, 1000 mL).

The dissolution profiles of the original and generic drugs in water with 1% SLS and pH 6.8 phosphate buffer are presented in Figure 2. The drug dissolved rapidly in 1000 mL of water with 1% SLS, recommended by the USP dissolution methods database for BIC tablets to facilitate sink conditions [32]. The percentage dissolved was 78% and 90% after 15 min in water with 1% SLS medium for the original and generic drugs, respectively (Figure 2A).

The drug release was low in the non-sink condition at pH 6.8, as expected (~7–8% in 1 h) (Figure 2B). Since the solubility of BIC (*pK_a_* = 11.49) is 12.95 × 10^−6^ mol/L (5.6 µg/mL) in water at 35 °C and is independent of pH [28]. The generic and the original drugs’ dissolution profiles were similar in water with 1% SLS (*f*_2_ = 58) and pH 6.8 medium (*f*_2_ = 94).

### 3.2. Biphasic Dissolution Test

**Figure 3:**
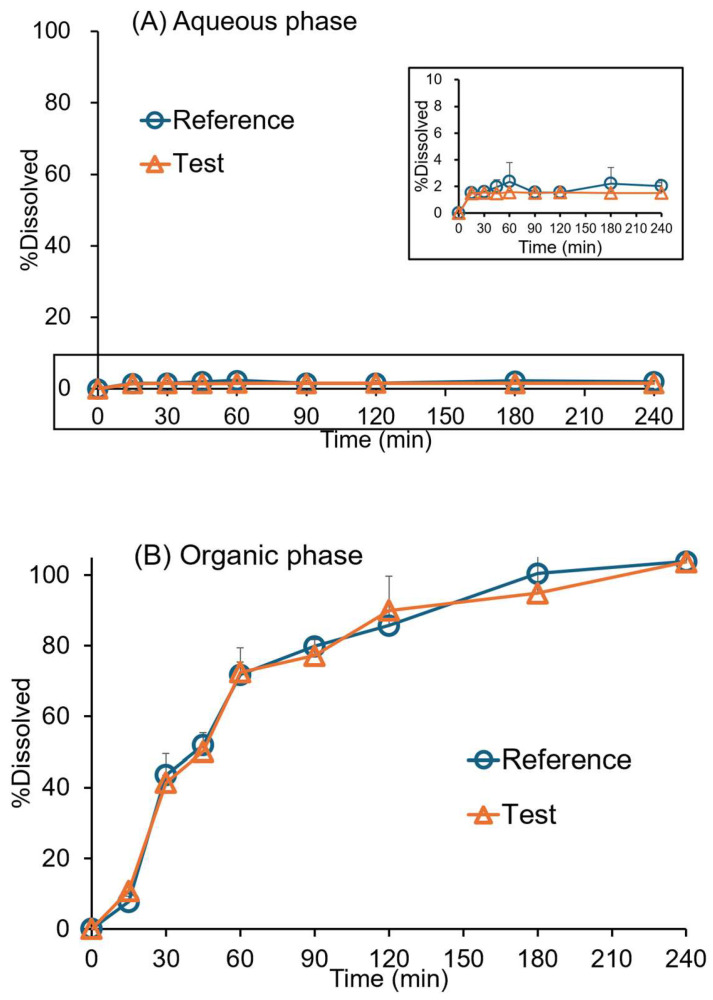
Dissolution profiles of 50 mg of BIC original (reference) and generic (test) tablets in the biphasic dissolution test: (**A**) pH 6.8 phosphate buffer, (**B**) octanol phases. Data were collected using the paddle-modified USP paddle (rotation speed = 50 rpm; 37 ± 0.5 °C; mean ± SD; *n* = 3).

The biphasic dissolution profiles of original and generic drugs in aqueous and organic phases are presented in Figure 3. The products exhibited similar profiles in the organic (*f*_2_ = 73.4) and aqueous (*f*_2_ = 98) phases. The dissolved BIC rapidly partitioned into the octanol phase between 15 min and one h. The partitioning is relatively slow after one h, exhibiting a time-dependent increase up to four h for the original and generic drugs.

### 3.3. Correlation Between Absorption and Partition Profiles

**Figure 4:**
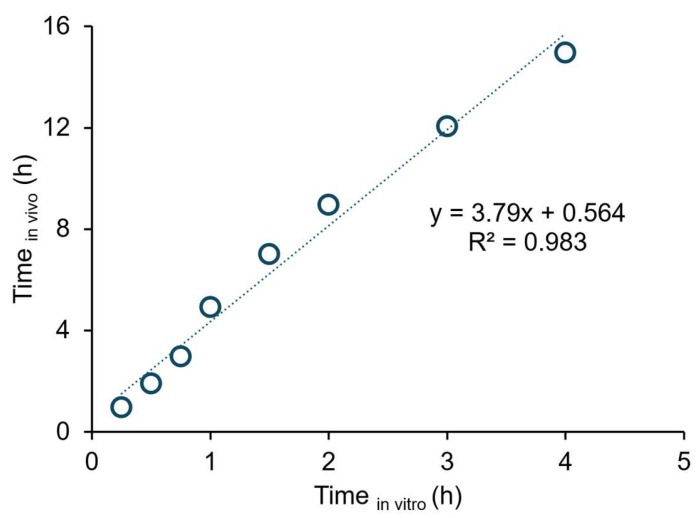
Levy’s plot for the original drug. Time_in vitro_ represents the sampling time points of the organic phase (15, 30, 45, 60, 90, 120, 180, and 240 min) in the biphasic dissolution test. Time_in vivo_ represents the first eight in vivo time points for the plasma profiles (1, 2, 3, 5, 7, 9, 12, and 15 h).

**Figure 5:**
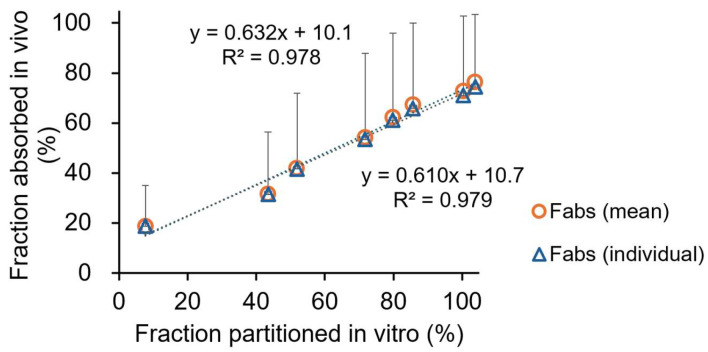
The point-to-point correlation between BIC’s fraction of the dose absorbed (*F_abs_*) and the extent of BIC partitioning into octanol for the original drug. (The triangles and circles show the individual and mean plasma data in healthy subjects, respectively. In vivo data were derived from the literature following administration of 50 mg single oral dose of the original drug to five healthy males [21].

A Levy’s plot was constructed to assess the relationship between dissolution and absorption (Figure 4). A good correlation was observed between in vitro and in vivo time points. The point-to-point correlation between BIC’s *F_abs_* and the extent of BIC partitioning into organic phase for the original drug is presented in Figure 5. A good correlation was evident between the extent of BIC partitioning into octanol and the *F_abs_* values calculated based on each subject and mean plasma data for the original drug (*r*^2^ = 0.979 vs. 0.978). The generic’s predicted mean *k_a_* value was 0.152 ± 0.119 h^−1^, of which 1.6 times is the original drug’s observed mean *k_a_* value (0.245 ± 0.233 h^−1^).

### 3.4. In Vivo Prediction with the Biphasic Dissolution Data

**Figure 6:**
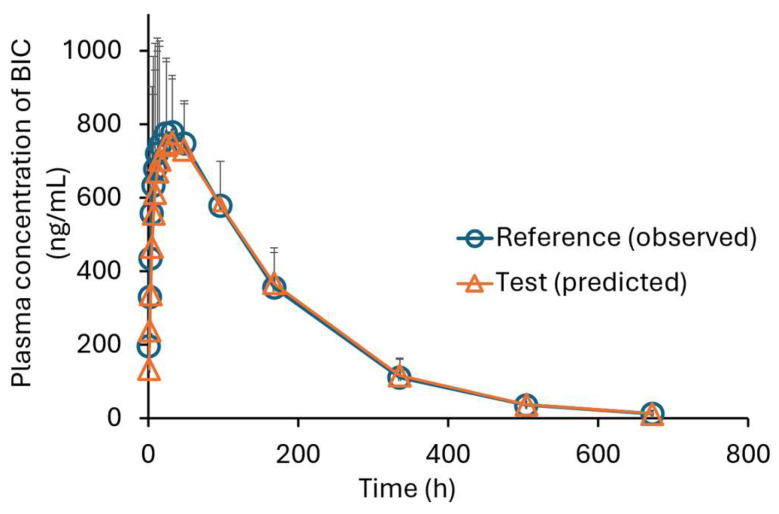
The mean plasma concentration–time profiles of BIC in five healthy males. (The circles are the observed profile for the original drug (reference) [21], and the triangles are the predicted profile for the generic drug (test), mean ± SD; *n* = 5).

**Figure 7:**
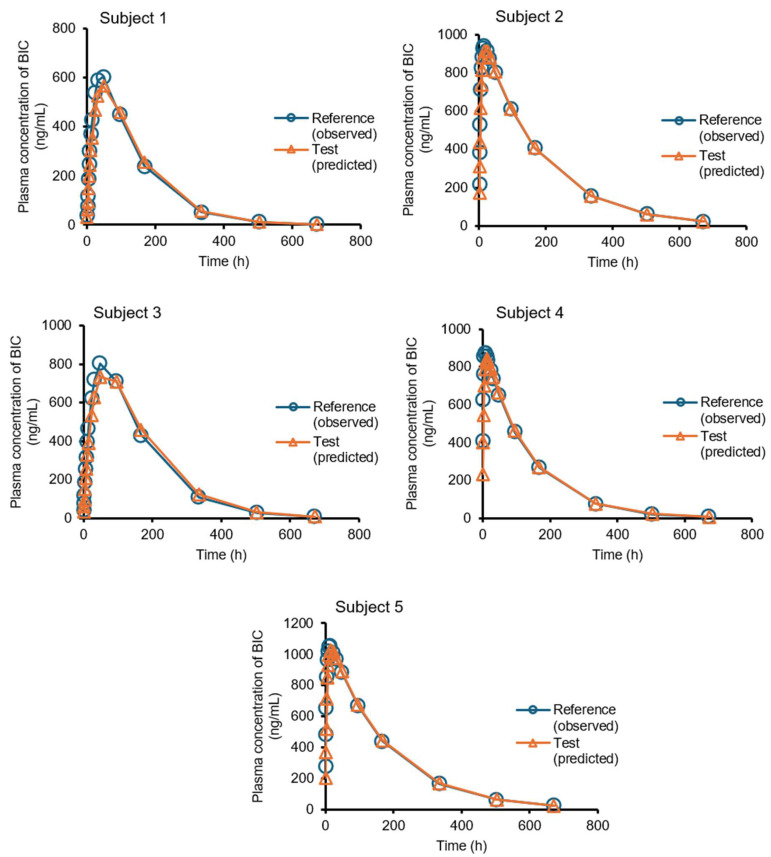
The plasma concentration–time profiles of BIC in five healthy male volunteers. (The circles are the observed plasma profile for the original drug (reference) [21], and the triangles are the predicted plasma profile for the generic drug (test)).

The predicted mean and single plasma profiles of the generic product and the original drug are presented in Figure 6 and Figure 7, respectively.

The *AUC* and *C_max_* values are presented in Table 2. The *AUC* and *C_max_* ratios for generic/original were 1.04 ± 0.01 (mean ± SD) and 0.951 ± 0.026 (mean ± SD), respectively.

## 4. Discussion

The present study characterized an IVIVC for BIC IR dosage forms with the biphasic in vitro dissolution test to forecast the drug’s BA in humans. The plasma profiles of the generic predicted using the in vitro dissolution data were compared with the original product’s profiles. Being a BCS Class II drug, BIC (MW: 430.373 g/mol) was selected because of its high permeability and low solubility [26,28]. In addition, the literature on human PK data of the original product enabled the conducting of an IVIVC [21].

BIC is a lipophilic compound with a *log P* of 2.92 and a *pK_a_* value of 11.49. The low solubility in aqueous media (~5 mg/L) is independent of the physiological pH values [20,28]. Based on the neutral characteristics, BIC can be classified as a BCS Class IIc drug [33,34]. Thus, the high unionized fractions of BIC at physiologically relevant pHs (~pH 1–7.4) and the enterohepatic circulation could explain its slow (time to reach *C_max_* (*t_max_*) = 15–48 h [21]) and extensive absorption after the oral dose. Furthermore, the dose linearity of BIC exists in the dose range of 10–50 mg. However, it deviates from linearity at the doses of 50 to 150 mg because of the saturation of the absorption process due to the poor water solubility of the drug [20].

This study performed single-phase dissolution experiments under both sink (water containing 1% SLS) and non-sink (phosphate buffer, pH 6.8) conditions to evaluate the original and generic products. The products exhibited expected profiles in the single-phase test conditions. However, the single-phase tests and surfactant media used to maintain sink conditions almost reduce the discriminative power of dissolution methods [7]. Considering BCS Class II drugs such as BIC exhibit limited dissolution in the GI tract but easily permeate the intestinal membrane, dissolution testing under non-sink conditions may offer a more physiologically relevant evaluation [35]. Moreover, compounds classified as BCS Class IIc exhibit solubility that remains largely unaffected by pH variations. As a result, predictive models that integrate physiological factors such as pH shifts and transit duration may offer limited additional value when estimating the in vivo performance of these drugs. Instead, incorporating an absorption phase into dissolution setups may provide a more effective strategy for developing methodologies that better simulate in vivo conditions [34]. More predictive in vitro dissolution approaches are necessary to properly evaluate the in vivo dissolution, absorption, and oral BA of BIC.

In this aforementioned context, the biphasic dissolution test could accurately represent in vivo dissolution and absorption processes of BIC formulations. The partitioning of BIC into octanol well reflected the intestinal absorption of BIC. The sink condition was kept in octanol (200 mL) during the experiment. The formulations exhibited similar profiles in the phosphate buffer and octanol phases. The partitioning of BIC into the organic phase was based on the time-dependent manner of the equilibrium of two phases, as previously reported [27]. The rapid partitioning of BIC into octanol was attributed to its fast release from the dosage form. Also, the concentration in the aqueous phase remained stable throughout the experiment, indicating that steady-state conditions were established. The organic phase maintained the sink conditions for continuous drug dissolution. The target release percentage of BIC in octanol was found to be approximately 70% in 1 h, 85% in 2 h, and 100% in 3 h for the bioequivalent IR formulations of BIC. The octanol phase served as a surrogate for the intestinal absorption of BIC. The partitioning of BIC into octanol in 4 h is well correlated with the absorption data in 15 h, describing its slow and extensive absorption after oral administration. For the deconvolution method, it was demonstrated that no significant difference exists between the mean and separate plasma data for establishing the point-to-point correlation between in vitro and in vivo (Figure 5).

The generic product showed a lower *k_a_* compared to the original drug (0.152 ± 0.119 h^−1^ vs. 0.245 ± 0.233 h^−1^). The discrepancy in *k_a_* may be related to the high variation of the absortion in five subjects. It can be attributed to the dissolution rate-limited absorption of BIC. Additionally, in vivo intestinal dissolution may be different than in vitro dissolution due to the GI physiology, active pharmaceutical ingredient, and formulation-related factors such as excipients and particle size for poorly soluble drugs. In fact, a minimum number of 12 evaluable subjects should be included in any bioequivalence (BE) study [36]. The predictive power of the present correlation requires in vivo data available for more subjects to assess BE of the two products.

Recent studies demonstrate a level A IVIVC among in vitro biphasic dissolution data and in vivo outcomes for the IR formulations of BCS Class II drugs in humans [27,37,38,39,40]. Al Durdunji et al. constructed a Level A IVIVC that complies with the FDA acceptance criteria using the biphasic dissolution test with the USP flow-through and apparatus II for deferasirox dispersible tablets. This discriminative system successfully simulated in vivo dissolution and absorption processes for the deferasirox formulations [37]. This pioneering Level A IVIVC study was subsequently followed by the others for BCS Class II drugs such as ritonavir, fenofibrate, aprepitant, celecoxib, itraconazole, nimodipine, and lamotrigine, assessing the in vivo predictive ability of the tests [27,38,39,40].

Overall, the present study demonstrated that the relationship between in vivo absorption and in vitro biphasic dissolution enabled forecasting the plasma profiles of BIC’s generic formulation based on the compartmental model for the first time. It was concluded that biphasic dissolution testing may present an in vivo predictive capability for the BCS Class II drugs such as BIC, characterized by pH-independent poor solubility and prolonged absorption phase. Accordingly, the biphasic in vitro dissolution system may serve as a time- and cost-saving tool in developing generic products.

## Figures and Tables

**Table 1:** Pharmacokinetic (PK) parameters derived from the literature following administration of 50 mg single oral dose of the original drug to healthy males [21].

Subject	*k_a_* (h^−1^) ^1,#^	*k_d_* (h^−1^) ^2,#^	V_d_/FF* ^3,#^ (mL)
1	0.0485	0.00910	55,948
2	0.247	0.00570	48,685
3	0.0321	0.00830	38,374
4	0.608	0.00750	54,048
5	0.290	0.00580	43,811
Mean ± SD ^4^	0.245 ± 0.233	0.00730 ± 0.0015	48,173 ± 7251
CV% ^5^	95.1	20.5	15.0

Note: ^1^ absorption rate constant; ^2^ elimination rate constant; ^3^ volume of distribution; ^4^ standard deviation; ^5^ coefficient of variation; **^#^** PK parameters calc. using $k_{a}=\frac{ln2}{t_{1/2}k_{a}}$, $k_{d}=\frac{ln2}{t_{1/2}k}$, and VdFF*=Dkd. AUC0→∞ where *t*_1/2_*k_a_* is the half-life of the absorption phase (h), *t*_1/2_*k* is the elimination half-life (h), *D* is the dose (µg), *AUC_0→__∞_* is the area under the plasma concentration–time curve from time zero to infinity (µg.h/mL), respectively.

**Table 2:** Bioavailability (BA) criteria for the generic (test) and original (reference) products in five healthy subjects.

Bioavailability Criteria	Test	Reference
	Mean ± SD ^1^	Mean ± SD
*AUC*^2^ (µg/mL.h)	157 ± 41.0	151 ± 40.3 °
*C_max_ *^3^ (ng/mL)	815 ± 175	855 ± 167 *

Note: ^1^ standard deviation; ^2^ area under the plasma concentration–time curve from time zero to infinity; ^3^ maximum plasma concentration; ° in vivo data were taken from the literature [21]; * in vivo data were derived from the literature following administration of 50 mg single oral dose of the original drug to five healthy males [21].

## Data Availability

The data presented in this study are available in this article.
